# Modes of immunosuppression in glioblastoma microenvironment

**DOI:** 10.18632/oncotarget.26643

**Published:** 2019-01-29

**Authors:** Suvi Lehtipuro, Matti Nykter, Kirsi J. Granberg

**Affiliations:** Kirsi J. Granberg: Faculty of Medicine and Health Technologies, Tampere University, Tampere, Finland; Science Center, Tampere University Hospital, Tampere, Finland

**Keywords:** glioma, immunosuppression, DNA methylation, IDH1, MHC-I

Recent success in checkpoint blockade therapies has provided a model example of targeted immunotherapies with strong and durable efficacy but only in a restricted responsive patient population [1, 2]. This suggests that checkpoint-mediated regulation is the basis of immunosuppression for those tumors, whereas the immune system is inactivated *via* other mechanisms, or their combinations, in other patients. A better understanding of these different routes to immunosuppression will enable us to effectively target immunosuppression in a personalized manner. This is also true for brain tumors, such as glioblastoma (GBM), which is an aggressive diffuse glioma type with an extremely poor prognosis [3]. Glioblastoma microenvironment typically includes high macrophage/microglia counts and few T cells [4]. While low in numbers, T cells are present in glioblastoma microenvironment, and T cell infiltration positively correlates with clinical outcome in these patients [4]. In principle, tumor-associated microglia and macrophages (TAMs) can attack malignant cells and have the capacity to present antigens to T cells upon activation, but they typically instead contribute to the immunosuppression and may even promote proliferation and progression of malignant cells [4, 5]. Consistently, they have been associated with poor patient prognosis.

The Cancer Genome Atlas (TCGA) recently analyzed cancer immune landscape across 33 tumor types and distributed tumors into six immune subtypes [6]. These subtypes are associated with differences in macrophages and lymphocytes, intratumoral heterogeneity, genomic landscape, cell proliferation, and prognosis [6]. This demonstrates that the immune system - tumor interaction can be categorized into relevant subtypes, which are not limited to a single tumor type. This analysis provides an overall framework for cancer immunology. Prevalent immune subtypes were clearly different in GBM and low-grade diffuse gliomas than in other tumors types [6], highlighting the immunological uniqueness of these tumors and the surrounding nervous tissue. Our recently published characterization of TCGA GBM cohort provides a complementary view to the immune landscape in GBM [7]. We used a data-driven approach to define gene clusters and sample groups which are relevant for glioblastoma immunology. These features were also associated with deconvolution-based estimates of relative immune cell type proportions as well as the clinical and genetic characteristics of the tumors. As we analyzed the data from a multitude of perspectives, we were able to investigate both the immune cell proportions and the nature of the immune response present in the tumor microenvironment, thus extending our understanding of immunosuppression in GBM.

**Figure 1:**
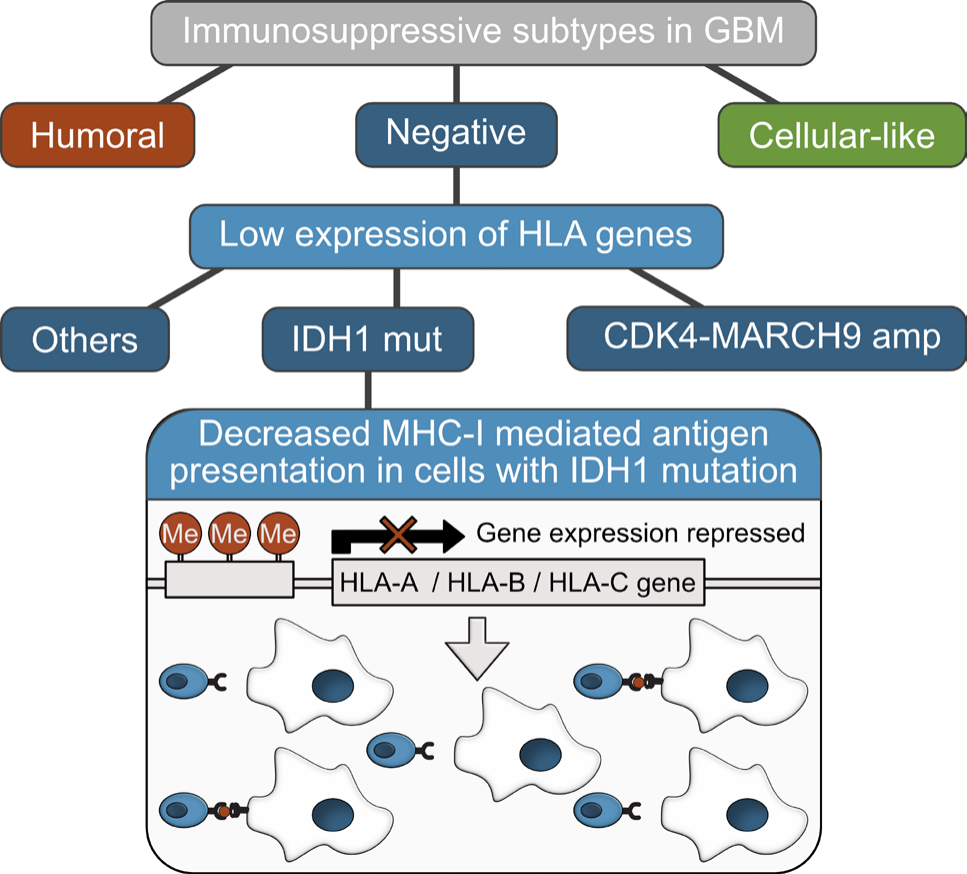
The possible mechanism of immunosuppression in samples with IDH1 mutation The expression of MHC I -type HLA genes is lowest in the negative subgroup and their DNA methylation levels are high in samples with an IDH1 mutation when compared to other samples. This suggests epigenetic silencing of HLA expression in IDH1 mutant tumors which potentially decreases MHC-I mediated antigen presentation in IDH1 mutant cells.

As a result, we observed high variation in the immune response -related gene signatures and estimated immune cell proportions, suggesting patient-to-patient variation in the modes of immune suppression. Sample clustering based on the discovered immune gene signatures identified three major tumor microenvironment subgroups: negative, humoral, and cellular-like (Figure 1). They can be considered to represent three general modes of immunosuppression in GBM, especially in respect to adaptive immune response.

Estimated immune cell proportions in GBM samples support our subgrouping, e.g., high B and CD4+ T cell proportions and low CD8+ T cell proportions were mainly observed in the humoral subgroup. The negative subgroup was characterized by the low activity of most immune response-related gene clusters and low immune cell recruitment when compared to other subgroups. All the IDH-mutated tumors were in this group, consistently with [6, 8]. Cellular-like subgroup contained samples with high activity of gene clusters ‘negative regulation of T cell activation, PD-L1’ and ‘gamma delta T cells’. However, CD8+ T cell recruitment to the tumor microenvironment, which should take place for proper anti-tumor immune response, was not significantly increased.

The obtained subgroups, cluster activities, and immune cell proportions showed associations with patient survival, GBM subtypes and typical genetic alterations in GBM (such as IDH mutation, inactivating NF1 alteration, and CDK4-MARCH9 locus amplification) [7]. NF1 inactivation was associated with activated innate immunity (increased granulocyte and TAM recruitment as well as TAM response) but not with our immune subgrouping, which is partly dependent on the adaptive immune response, as well. Interestingly, most of the strong genetic associations distinguished negative subgroup from the others but did not make a clear distinction between humoral and cellular subgroups. Although the small size of the humoral subgroup can contribute to the results, this also suggests that the studied genetic features are influencing more the level of adaptive immune response than the type of response. In general, MHC I -mediated antigen presentation, which is needed for CD8+ T cell activation, appears to be decreased in the negative group (Figure 1). For example, different MHC I -type HLA genes have the lowest expression in the negative subgroup and the highest in the cellular-like group [7]. The lowest expression of these HLA genes is in the samples with either IDH1 mutation or amplification of CDK4-MARCH9 locus. Furthermore, HLA genes are significantly more methylated in samples with IDH1 mutation than in other tumors, suggesting epigenetic silencing of HLA expression in IDH1 mutant tumors (Figure 1). This also seems to be the case, as HLA protein expression was lowest in an IDH-mutant cell line among diffuse glioma cell lines and methyltransferase inhibition was able to release this suppression and increase HLA expression in our experiments [7]. This also has clinical implications, as improved antigen presentation might be a potential intervention option for cases in the negative subgroup. In the future, revealed different modes of immunosuppression in GBM together with associated molecular features can guide patient stratification and therapeutic interventions.
